# Annual Commencement of the Baltimore College of Dental Surgery

**Published:** 1875-02

**Authors:** 


### EDITORIAL, ETC.

---

*Annual Commencement of the Baltimore College of Dental Surgery.*—The *Thirty-fifth* Annual Commencement of this institution will take place on the evening of February 25th, 1875, at the Concordia Opera House, Baltimore City, and the indications are that the Graduating Class will exceed in number that of last session.

The Alumni and Friends of the College are very cordially invited to be present, not only at the commencement exercises, but during the final examination of the candidates for graduation, which takes place during the week preceding the Commencement.

The Faculty feel assured that the course of study and requirements, as well as the result of the examinations, will convince all honest minds, that not only a very marked improvement has been brought about by the officers of this College, in their endeavors to elevate the standard of dental education, within the past five years, but that this, the oldest Dental College in the world, still maintains its world wide reputation, and while it is far superior to many of recent growth, is second to none in this or any other country.

The Faculty invite all interested in dentistry to visit this institution in order that they may, after like visits paid to others, be able to make a fair comparison, and no longer be deceived by false statements which generally accompany incompetency and failure.

As an evidence of the desire of the Faculty of the Baltimore College of Dental Surgery to do every thing in their power to advance the interests of dentistry, and maintain and elevate the status of their institution, they take pleasure in announcing that as soon as the present session terminates, this College will be removed to the handsome four story building with mansard roof, which has just been completed for the purpose, on the South-East Corner of Lexington and Eutaw Streets, one of the best locations in the city of Baltimore; and they can now truthfully say that it will be the most elegant and best arranged Dental College in the world.

Its location possesses every advantage for securing a larger Infirmary practice than even now exists, and the friends of the College are invited to visit the new building and judge for themselves.

It will also be gratifying for them to learn that the number of students in attendance at the lectures of the present session is larger than that of last session.

